# Determination and Pharmacokinetics of Omeprazole Enantiomers in Human Plasma and Oral Fluid Utilizing Microextraction by Packed Sorbent and Liquid Chromatography-Tandem Mass Spectrometry

**DOI:** 10.1155/2021/8845139

**Published:** 2021-01-19

**Authors:** Hytham Ahmed, Abdel-Aziz Wahbi, Hatem Elmongy, Ahmad Amini, Hirsh Koyi, Eva Branden, Mohamed Abdel-Rehim

**Affiliations:** ^1^Department of Pharmaceutical Analysis, Menoufia University, Menofia Governorate, Egypt; ^2^Department of Pharmaceutical Analytical Chemistry, University of Alexandria, Alexandria 21521, Egypt; ^3^Swedish Drug Agency, Uppsala, Sweden; ^4^Department of Respiratory Medicine Gävle Hospital and Centre for Research at Uppsala University/County Council of Gävleborg, Gävle, Sweden; ^5^Karolinska Institute, Solna SE17176, Sweden

## Abstract

In the present work, the determination of omeprazole (OME) enantiomers in oral fluid and plasma samples was carried out utilizing microextraction by packed sorbent (MEPS) and liquid chromatography-tandem mass spectrometry. A chiral column with cellulose-SB phase was used for the first time for enantiomeric separation of OME with an isocratic elution system using 0.2% ammonium hydroxide in hexane-ethanol mixture (70 : 30, v/v) as the mobile phase. OME enantiomers were determined utilizing a triple quadrupole tandem mass spectrometer in positive ion mode (ESI+) monitoring mass transitions: *m*/*z* 346.3 ⟶ 198.0 for OME and *m*/*z* 369.98 ⟶ 252.0 for internal standard. The limits of detection and quantification of the present method for both enantiomers were 0.1 and 0.4 ng/mL, respectively. The method validation provided good accuracy and precision. The matrix effect factor was less than 5%, and no interfering peaks were observed. The interday precision values ranged from 2.2 to 7.5 (%RSD), and the accuracy of determinations varied from −9.9% to 8.3%. In addition, the pharmacokinetics (PK) of omeprazole enantiomers in healthy subjects after a single oral dose was investigated. (S)-Enantiomers showed higher levels than (R)-enantiomers throughout 24 h. It was found that the mean maximum concentrations of (R)- and (S)-omeprazole in plasma samples were about two times higher than in oral fluid.

## 1. Introduction

Omeprazole (OME) is the first proton pump inhibitor (PPI) that acts by blocking the H+- K + ATPase enzymes (proton pumps) irreversibly. OME is indicated in treatment of all acid-related diseases, where the target of treatment is to reduce gastric acid secretion, such as dyspepsia, peptic ulcer, gastric ulcer, and gastroesophageal reflux disease [1]. It is a chiral drug but used as a racemic mixture and pure enantiomer for medicinal purposes [2]. Commonly, the pharmacodynamics and pharmacokinetics (PK) of a chiral drug are potentially different between the optical isomers. Therefore, it is important to study the individual enantiomers to get clear knowledge about pharmacokinetics and pharmacodynamics of any chiral drug. In OME drug, the (S)-enantiomer (esomeprazole) showed better effect, and it was formulated as an enantio-pure drug by AstraZeneca under the commercial name of Nexium® [3–5]. Solid-phase extraction (SPE) is the mainly employed sample preparation technique for extracting OME from plasma [6–9]. In addition, liquid-liquid extraction (LLE) [10] and 96-well LLE [11] have been applied for OME measurement in biological samples. However, SPE provides some advantages, such as selectivity and efficiency, over other techniques, especially LLE [12, 13]. Moreover, achiral-chiral column-switching technique has been used for online precolumn extraction of OME [14]. However, there is an increasing demand for miniaturized and automated sample preparation techniques which are simple, cheap, and provide acceptable drug recovery and selectivity [15].

Microextraction by packed sorbent (MEPS) is a miniaturized SPE technique, in which the volumes of sample and solvents is highly reduced. MEPS is efficient for the removal of contaminants from samples. In MEPS, the packed sorbent is integrated directly into the syringe via sampling needle which could be used several times for biological samples instead of single-use SPE cartridges [16], can be fully automated, and be used for small-volume samples [16–21].

As oral fluid is easier to handle and is less complex than the plasma matrix, it has been a promising matrix for drug analysis and pharmacokinetic studies [22–27]. Oral fluid samples can be easily collected from infants, disabled, or anxious patients [28]. In comparison with plasma, oral fluid can provide shorter sampling time, more simple and noninvasive sampling strategy. Furthermore, daily secretion of human oral fluid is too high (up to 1.5 L) which enables sample collection at short intervals [29, 30]. Therefore, oral fluid can be considered as an ideal biological fluid for drug monitoring.

OME enantiomers have been separated by using chiral columns with polysaccharide-based stationary phases formed of carbamate derivatives of cellulose (Chiralpak AD column, Chiralcel OJ column, Chiralpak AS column, and Chiralcel OD column) [7, 8, 13] or amylose [14, 31] as well as protein-based chiral stationary phase (bovine serum albumin) [6].

Several studies have been conducted for investigating the pharmacokinetic parameters of OME and its enantiomers in human plasma after single oral dose [32] or repeated oral doses [33]. Oral fluid samples provide a safe solution for sample collection procedures. The unbound drug molecules, being responsible for the therapeutic activity of the administered drug, are distributed to the oral fluid [34].

The present work describes an enantioselective chromatographic method with mass spectrometric detection for the separation and quantification of OME enantiomers in plasma and oral fluid samples using MEPS for sample preparation and chiral column cellulose-SB tris(3,5dimethylphenylcarbamate) for the chiral separation. The developed method aims to provide a reliable determination of OME enantiomers in oral fluid as an alternative specimen to plasma to monitor the therapeutic concentrations of the drug. To our knowledge, the present study is the first study for the analysis of OME enantiomers in human oral fluid samples, using a cellulose-SB-based stationary phase.

## 2. Materials and Methods

### 2.1. Chemicals

OME racemate, (R)-OME, (S)-OME, and (S)-lansoprazole (I.S) were purchased from Sigma-Aldrich (Steinheim, Germany). HPLC-grade hexane, ethanol, formic acid, and ammonium hydroxide were purchased from Merck (Darmstadt, Germany). A Milli-Q Plus water purification system from Millipore Corporation (Bedford, USA) was used for water purification.

### 2.2. Preparation of Standards and Quality Control Samples

Two stock solutions (5 *µ*g·mL^−1^, each) were prepared in ethanol (one to prepare calibration standards and one for the quality control (QC) samples), and the standards and QC samples were prepared in blank pooled human plasma (heparin) and oral fluid samples (from six different subjects). The stock solutions showed stable for at least one month at 4°C. The concentration of the standard samples in plasma was in the range of 25–600 ng/mL and in oral fluid was 25–300 ng/mL. QC samples were prepared in plasma and oral fluid in three concentration levels: low (QCL), medium (QCM), and high (QCH) as reported in Table 1.

### 2.3. Instrumentation

The liquid chromatography system (LC) consists of two pumps, Shimadzu LC-10ADvp, (Kyoto, Japan), an autosampler (CTC-Pal, Analytics AG, Zwingen, Switzerland), and a 50 *µ*L sample loop. The used chromatographic column was a CHIRAL ART cellulose-SB (150 × 4.6 mm, 5 *μ*m particle size) and was obtained from YMC Europe GmbH (Dinslaken, Germany). Different mobile phase composition utilizing ethanol and hexane with different additives (formic acid and ammonium hydroxide) was investigated. The best chromatographic conditions consisted of 0.2% ammonium hydroxide in n-hexane/ethanol (70 : 30, v/v). The mobile phase flow rate was at 0.8 mL/min with split of 1 : 1 before the MS interface. A 50 *μ*L sample volume was injected. eVol sampling device with MEPS syringe and C8 sorbent pins were supplied by SGE Analytical Science (Melbourne, Australia).

Electrospray ionization in positive ion mode (ESI+) was performed using a triple quadrupole mass spectrometer detector (Quattro-micro, Waters, Manchester, UK). 150 and 400°C were the MS source block and desolvation temperatures, respectively. Nitrogen gas was utilized not only for drying (950 Lh^−1^) but also for nebulization (60 Lh^−1^). Argon gas was utilized as collision gas (collision energy 10 eV for OME and 20 eV for lansoprazole). OME enantiomers analysis in plasma and oral fluid samples was performed by using LCMS/MS with multiple reaction monitoring (MRM) of the transitions: *m*/*z* 346.30 > 198.05 for OME and *m*/*z* 369.98 > 252.02 for I. S. utilizing a dwell time of 0.2 sec/transition. Peak-area ratios (OME/I. S.) were used for all calculations. Data analysis was done by using MassLynx software (version 4.1).

### 2.4. Sample Preparation

The QC samples in plasma and oral fluid were prepared and stored at −20°C until the analysis, while the standard calibrations were prepared freshly for each validation assay. A 100 *μ*L of each sample was mixed with a 100 *μ*L of the I. S (20 *µ*g mL-1 in methanol), and then the samples were diluted with water (1 : 4) and centrifuged for few minutes (1–3 min). Thereafter, extraction utilizing a programmed eVol device with MEPS syringe contains C8 sorbent. The sorbent was conditioned using 100 *µ*L ethanol followed by 100 *µ*L of water. The sample was drawn 6 times for preconcentration of the analytes. Then, the sorbent was washed by 5% methanol in water (2 × 100 *μ*L) two times to take away the interfering materials in the samples. A 500 *µ*L ethanol (2 × 250 *μ*L) was utilized for the analyte and I.S. elution. The samples were then subjected to evaporation until dryness under nitrogen, and the residues were reconstituted with 150 *µ*L ethanol. The MEPS sorbent was reused after washing 3-4 times with water and 4-5 with ethanol to overcome carry over.

### 2.5. Method Validation

The validation of the proposed method was carried out according to FDA guidelines [35] including accuracy, precision, matrix effect, selectivity, linearity, limits of detection and quantitation, recovery, and robustness.

Quality control samples at three different levels: low (QCL), medium (QCM), and high (QCH) (*n* = 6) were used for the determination of precision and accuracy of the method. For intraday precision and accuracy, a single analytical batch was analyzed. On the other hand, three different batches with three triplicates were analyzed for the evaluation of the interday precision and accuracy. The accepted data for the precision with relative standard deviation (RSD) should be within ±15%, and the accuracy with relative error (RE) from the nominal values should not exceed 15% and 20% for lower limit of quantitation (LLOQ).

The calibration curve for plasma ranged from 25 ng/mL to 600 ng/mL (25, 50, 100, 150, 200, 250, 300, and 600 ng/mL), while for oral fluid, it was in the range of 25–300 ng/mL (25, 50, 100, 150, 200, 250, and 300 ng/mL). Each back calculated standard concentration should not be more than two-third of the points to be accepted with %RSD≤15% except lowest concentration (≤20%).

The stability of OME in plasma and oral fluid was performed using triplicates of the QC solutions. Four types of stability studies were done for QC samples. First type was short-term stability by defrost the samples at room temperature for eight hours before analysis time; the second type was long-term stability via keeping the samples at −20°C for fifteen days before analysis time; the third type was three freeze-thaw stability cycles by leaving the iced samples at −20°C to melt at room temperature. After that, samples were restored at −20°C, and this procedure was repeated three times before analysis time, and finally, the last type was postpreparative stability of the processed samples by keeping them for 24 h at 4°C before analysis time. If assay results were within the acceptable limits of accuracy (±15%) and precision (15%), then samples could be considered to be stable.

### 2.6. PK Analysis

The PK parameters were calculated from both oral fluid and plasma samples using the concentration-time profile data for each enantiomer. *C*_max_ and *T*_max_ were calculated directly from the concentration-time plot of the individual enantiomer. The elimination half time (*t*_1/2_) and the elimination rate constant (*k*_e_) were estimated from the terminal slope of the plasma/fluid concentration-time profile based on first-order kinetics.

## 3. Result and Discussion

### 3.1. Method Development

**Figure 1:**
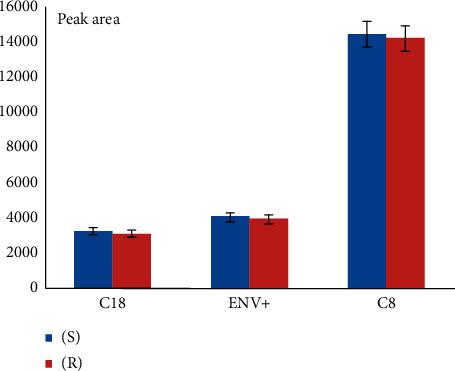
Effect of sorbent type on extraction efficiency (sample conc. 0.01 *µ*g·mL–1).

**Figure 2:**
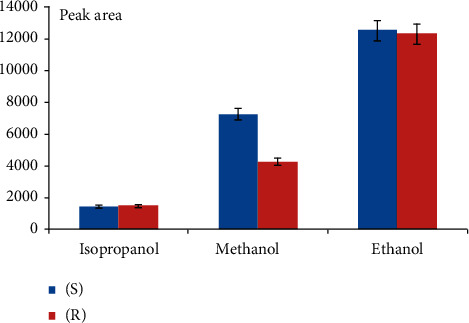
Effect of elution solvent on extraction efficiency (C8, sample conc. 0.05 *µ*g·mL^−1^).

The extraction of OME enantiomers from plasma and oral fluid samples was achieved using MEPS eVol sampling device. Different factors affecting extraction efficiency such as sorbents (C8, C18, and polystyrene), washing, and eluting solutions (methanol, ethanol, and isopropanol) were investigated. Best results were obtained with C8 as sorbent (Figure 1), water as washing solution, and ethanol as eluting solvent (Figure 2).

**Figure 3:**
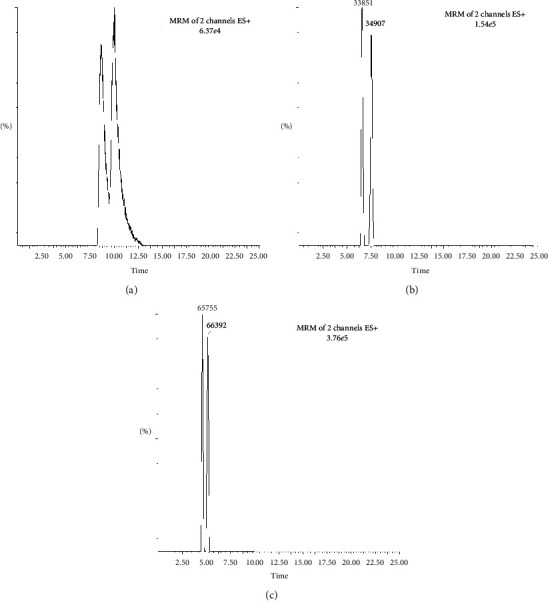
Effect of mobile phase composition on the separation of OME enantiomers: (a) hexane + isopropanol (80 : 20) as mobile phase, (b) hexane + ethanol (80 : 20), and (c) hexane + ethanol + NH_3_OH (70 : 30 : 0.2 v/v).

The chromatographic separation of OME enantiomers was achieved using chiral-SB column packed with cellulose tris-(3,5-dimethylphenylcarbamate) immobilized on silica gel as the chiral selector. Hexane containing different volumes of ethanol or isopropanol was investigated as a mobile phase. Different mobile phase composition consisting of ethanol and hexane with different additives (formic acid and ammonium hydroxide) was investigated. It is known that the addition of formic acid in the mobile phase results in good ionization in ESI interface, but in our case, it did not work well and it resulted in poor peak shape and poor enantio-separation. The addition of ammonium hydroxide on the other hand generated Gaussian peak shape and good MS signal for both the OME enatiomers and internal standard. Thus, the best result was obtained using a mobile phase consisting of n-hexane/ethanol (70 : 30, v/v) and ammonium hydroxide (0.2% v/v). The presence of ammonium hydroxide as a basic modifier in the mobile phase improved the enantiomeric selectivity and peak symmetry (Figure 3). The mobile phase flow rate was 0.8 mL·min^−1^, and a baseline separation between all analytes was achieved within 10 min of the run.

The triple quadrupole mass spectrometry was employed for the identification of OME enantiomers. For both enantiomers the precursor and product ions were appeared at 346.30 and 198.05 *m*/*z* values, while the corresponding ions for the internal standard were detected at 369.98 and 252.02 *m*/*z*, respectively.

### 3.2. Method Validation

The validation of the method was run as described previously including linearity, limit of detection (LOD), lower limit of quantification (LLOQ), accuracy, precision, recovery, matrix effects, selectivity, and carry over for determination of OME in human plasma and oral fluid samples in line with FDA guidelines. The calibration curves were prepared in the range of 25–600 ng·mL^−1^ and 25–300 ng·mL^−1^ in human plasma and oral fluid samples, respectively, with LLOQ of 25 ng/mL and a quadratic equation weighted with 1/*x* was used for the calculations. The calculated limits of detection (S/N ≥ 3) and quantitation (S/N ≥ 10) for both enantiomers in plasma and oral fluid were 0.1 and 0.4 ng/mL, respectively. The accuracy and precision were determined by the analysis of QC samples (QC low, QC medium, and QC high) as mentioned in Table 1.

#### 3.2.1. Calibration

**Figure 4:**
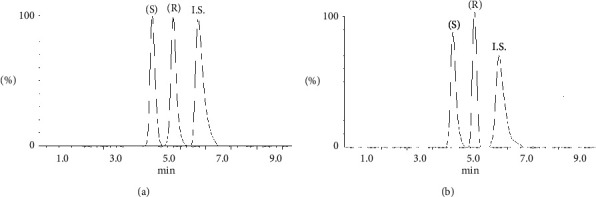
LC-MS/MS chromatograms of OME enantiomers in plasma (a) and oral fluid sample (b).

The peak area ratio of each enantiomer to I. S. was plotted against the corresponding concentration for construction of calibration curves for plasma and oral fluid samples. The quality-control samples were treated in the same way as the standards. Quadratic equation was used for the standard curve due to the complexity of the plasma and oral fluid matrices. Using oral fluid and plasma, a close relationship between concentration and peak area ratio (OME/I. S.) was observed in the concentration range 25–600 ng/mL and 25–300 ng/mL in plasma and oral fluid, respectively. The correlation coefficient (*R*^2^) values obtained for plasma and oral fluid matrices were ≥0.999.

(S)-Lansoprazole was used as internal standard, and the LLOQ was set to 25 ng/mL. A good correlation between peak area ratios and analyte concentrations was obtained which demonstrated reliability of the method.  Figure 4 shows LC-MS/MS chromatograms of OME in plasma and oral fluid.

#### 3.2.2. Accuracy and Precision

Table 1 reports the accuracy and precision data from the QC samples in plasma and oral fluid samples. Precision was calculated as the percentage of RSD, and the accuracy was calculated as the percent deviation of the obtained value from nominal value. The method accuracy and precision were within the accepted range, i.e., the accuracy and the precision values for plasma samples ranged from −1.9% to −10.3% and between 2.2 and 5.8%, respectively, for both intra- and interday assays. For oral fluid samples, the accuracy values varied between −9.9% and 8.3%, and the precision ranged from 4.3 to 7.5% for both intra- and interday assays.

#### 3.2.3. Extraction Recovery

The average absolute recovery for each enantiomer in both plasma and oral fluid samples using QC samples was 95%.

#### 3.2.4. Matrix Effect and Selectivity

Matrix effects of the tested biological fluids samples of OME enantiomers were shown to be within 95% and 103%, and thus no significant ion suppression was observed.

For the method selectivity, blank plasma and oral fluid samples from different subjects (*n* = 6) were analyzed. The results showed that no interfering peak was detected at the elution positions of at the enantiomer's and I. S.

#### 3.2.5. Stability

The stability studies were conducted analyzing each QC sample in triplicate. No significant change in the enantiomers concentration in short- and long-term being indicated by the mean recovery values was observed. Also, there was no effect on the quantification of target analyte in the three freeze-thaw cycles as well. In addition, the samples were assayed after 24 h at 4°C (postpreparative stability). The obtained data indicated that both used biological samples could be analyzed under such laboratory conditions and after storage at −20°C for up to two weeks without significant change in the drug chemistry or quantity (Table S1).

### 3.3. Comparison of Developed Method for OME Analysis

Several methods for extraction and analyzing of OME have been developed. In this part, ability of the developed method was compared with other methods, and the results of this part are presented in Table 2.

### 3.4. Method Application to PK Study of OME in Healthy Individuals

The PK study of OME enantiomers was performed in a group of 4 healthy subjects. The patient's samples were provided by the center of clinical research, Gavle hospital, Sweden. The subjects were 3 females and one male within an age range of 32–63 years old and a weight varying from 60 to 95 kg (Table 3). The study addresses the PK parameters based on a single oral administration of 20 mg OME tablets (racemate) to each volunteer. Blood and saliva samples were collected from each individual throughout 24 h after administration of the drug.

The OME enantiomers in human plasma and saliva samples were analyzed by the present method. The PK characteristics being expressed by its parameters, such as elimination rate constant (*k*_e_) and half-time (*t*_1/2_), were then estimated using the concentration-time profile data for each enantiomer. *C*_max_ and *T*_max_ were estimated directly from the concentration-time curves.

The PK parameters were evaluated for each enantiomer in the four healthy persons by calculating the mean values (Table 4). The results showed that the (S)-enantiomer has higher *C*_max_ and exposure as expressed as the area under the concentration-time curve (AUC) PK values compared to that found for the (R)-enantiomer ((R)-OME) by approximately 1.5 and 1.3-fold, respectively. *C*_max_ for both enantiomers reached 2.5 h after drug administration (*T*_max_) (Figure S1). The elimination rate of the (R)-enantiomer was slightly higher than that of the (S)-enantiomer. The half-time (*t*_½_) of the S-enantiomer was approximately 0.7 h compared to that obtained for the R-enantiomer. The higher oral clearance rate (CL/*f*) of the (R)-enantiomer provides a proof of the stereoselective metabolism. The (Vd/*f*) values of both enantiomers were approximately similar indicating high tissue distribution of both forms. The (S)-enantiomer showed higher *C*_max_ than that of (R)-OME with similar AUC0-24 values (Table 4). Both enantiomers showed faster *T*_max_ values in saliva compared to that seen in plasma, while both enantiomers attained higher AUC and *C*_max_ values in plasma than that in saliva.

## 4. Conclusion

A new method based on LC-MS/MS has been developed and validated for the assay of the enantiomers of OME in human plasma and oral fluid samples. Both plasma and oral fluid samples were treated with MEPS technique before analysis. Quantitative analysis of OME enantiomers in both matrices were achieved successfully with good accuracy and precision. The method demonstrated to be an excellent method for the chiral separation of OME enantiomers as well as for the monitoring of OME in plasma. Therefore, the method was used for the PK study of the omeprazole enantiomers.

The evaluation of the PK parameters in oral fluid for omeprazole showed poor correlation to those in plasma. However, the use of oral fluid serves as a fast and easy sampling medium for screening of the drug's therapeutic levels. On the other hand, possible enzymatic actions in the oral fluid and the intersubject variations in the enzymatic composition of oral fluid *s* can contribute to the variation in the drug levels that requires further investigation.

## Figures and Tables

**Table 1:** Accuracy and precision of QC samples in human plasma and oral fluid.

Sample matrix drug		QC (ng/mL)	Intraday (*n* = 6)		Interday (*n* = 18)	
			RSD (%)	Accuracy (%)	RSD (%)	Accuracy (%)
Plasma	(R)	75	5.7	−8.8	4.9	−5.7
		250	2.4	−5.9	2.2	−6.1
		500	5.1	−10.3	6.6	−2.4
	(S)	75	2.3	−3.5	4.4	−4.4
		250	5.0	−7.0	3.2	−1.9
		500	5.8	−7.1	5.6	−7.0

Oral fluid	(R)	75	5.3	−5.5	5.3	−3.5
		150	4.3	−3.7	4.5	8.3
		250	4.6	−4.0	3.5	−5.4
	(S)	75	5.6	5.0	7.5	4.8
		150	4.5	−4.7	5.3	−9.9
		250	4.2	6.9	5.0	−5.7

**Table 2:** Determination of OME with different methods.

Method	Linear range (ng/mL)	Matrix (OM form)	Sample preparation method	Extraction efficiency (%)	Ref.
RP-HPLC	2–2000	Human plasma (racemate)	LLE	84	[10]
HILIC-MS/MS	2–1000	Human plasma (racemate)	LLE	87–98	[11]
UHPLC-MS/MS	10–500	Rat plasma (racemate)	Protein precipitation and LLE	87	[36]
LC-UV	50–1000	Human plasma (enantiomers)	SPE	93–94	[37]
LC-MS/MS	1.25–2500	Human plasma (enantiomers)	Protein precipitation (PP)	105–106	[38]
LC-MS/MS	25–500	Human plasma, oral fluid (enantiomers)	MEPS	94–98	Present study

**Table 3:** The physical data of selected volunteers.

Subject no.	Sex	Weight	Age
1	Male	80	56
2	Female	95	59
3	Female	60	63
4	Female	70	32
	Average	76.25	52.5

**Table 4:** Main pharmacokinetic parameters of OME enantiomers in plasma and oral fluid following single oral dose of 20 mg to healthy volunteers (*n* = 4).

Parameter	Plasma		Oral fluid	
	(R)-OME	(S)-OME	(R)-OME	(S)-OME
*C*_max_ (ng·mL^−1^)	62.44	97.24	40.01	43.61
*T*_max_ (h)	2.50	2.50	2.00	2.00
*k*_el_ (h^−1^)	0.267	0.207	1.811	1.151
*t*_1/2_ (h)	2.59	3.35	0.58	0.60
AUC_0−24_	384.86	510.80	125.99	130.66
AUC_0−∞_	411.72	544.25	128.59	134.79
CL/*f* (L·kg^−1^·h^−1^)	0.631	0.477	1.944	1.855
VD/*f* (L·kg^−1^)	2.361	2.306	1.073	1.611

## Data Availability

The data used to support the findings of this study are available in supplementary information files.

## Supplementary Materials

Supplementary Materials Figure S1The mean plasma concentration-time curve of (a)(R)-omeprazole and (b)(S)-omeprazole in females compared to male volunteers after 24 h of the administration of a single oral tablet containing 20 mg of omeprazole racemate. Table S1Stability data of omeprazole enantiomers in plasma and saliva.
