# Supplementary material for: Determination and Pharmacokinetics of Omeprazole Enantiomers in Human Plasma and Oral Fluid Utilizing Microextraction by Packed Sorbent and Liquid Chromatography-Tandem Mass Spectrometry
Source: Int J Anal Chem. 2021 Jan 19;2021:8845139. doi: 10.1155/2021/8845139 (PMC7837791; doi:10.1155/2021/8845139)
Supplement: Supplementary Materials — Figure S1The mean plasma concentration-time curve of (a)(R)-omeprazole and (b)(S)-omeprazole in females compared to male volunteers after 24 h of the administration of a single oral tablet containing 20 mg of omeprazole racemate. Table S1Stability data of omeprazole enantiomers in plasma and saliva. [file 8845139.f1.pdf]

Figure S1

The mean plasma concentration - time curve of (A) (R)-omeprazole and (B) (S)-omeprazole in females compared to male volunteer after 24 h of the administration of a single oral tablet containing 20 mg of omeprazole racemate.

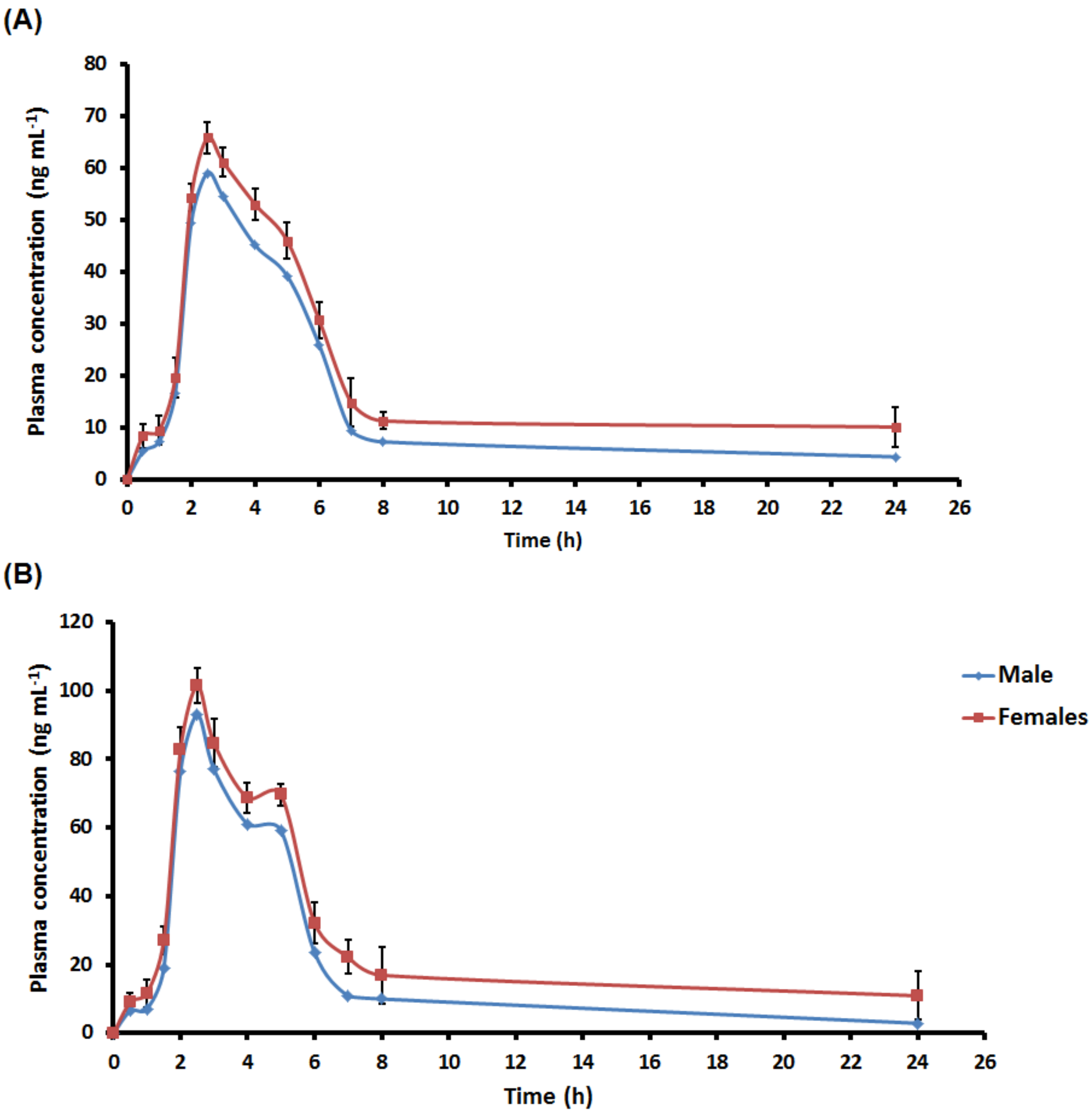

Table S1

Stability data of omeprazole enantiomers in plasma and saliva

Stability condition	Quality control (ng mL^{-1})	Plasma				Saliva			
		Accuracy (% deviation)		Precision (% RSD)		Accuracy (% deviation)		Precision (% RSD)	
		(R)-omeprazole	(S)-omeprazole	(R)-omeprazole	(S)-omeprazole	(R)-omeprazole	(S)-omeprazole	(R)-omeprazole	(S)-omeprazole
Short term stability	75	-2.6	-3.3	2.7	3.7	-1.6	-2.7	1.9	3.0
	250	-2.6	-4.5	3.1	4.8	-4.0	-3.2	4.8	3.8
	500	-1.3	-3.8	1.7	4.0	-1.5	-5.1	1.7	5.7
Long term stability	75	-3.6	-4.8	3.8	5.0	-6.2	-5.5	6.8	5.8
	250	-6.1	-6.0	6.5	6.4	-4.0	-5.0	4.3	5.5
	500	-3.9	-5.8	4.0	6.0	-3.0	-1.3	3.2	1.6
Freez-thaw stability	75	-7.4	-5.0	8.0	5.1	-5.6	-4.1	5.9	4.4
	250	-3.1	-3.9	4.1	4.2	-3.6	-6.2	3.8	6.5
	500	-6.7	-6.9	7.0	7.2	-3.2	-2.9	3.6	3.0
Post-preparative stability	75	-6.2	-3.1	6.6	3.6	-7.2	-4.7	7.4	5.1
	250	-3.8	-4.7	4.0	5.1	-3.3	-5.2	3.9	5.8
	500	-4.6	-2.6	4.9	2.9	-2.1	-3.3	2.7	4.0
